# Management and outcomes for older women with early breast cancer treated with primary endocrine therapy (PET)

**DOI:** 10.1016/j.breast.2024.103768

**Published:** 2024-07-08

**Authors:** Thomas Hubbard, Georgia Wright, Jenna Morgan, Charlene Martin, Stephen Walters, Kwok-Leung Cheung, Riccardo Audisio, Malcolm Reed, Lynda Wyld, the Bridging the Age Gap Trial Management Group

**Affiliations:** a Royal Devon University Hospital NHS Trust, Exeter, UK; b Faculty of Health Sciences, University of Exeter, Exeter, UK; c Division of Clinical Medicine, University of Sheffield, Sheffield, UK; d School for Health and Related Research (ScHARR), University of Sheffield, Sheffield, UK; e School of Medicine, University of Nottingham, Royal Derby Hospital, Derby, UK; f University of Gothenberg, Sahlgrenska Universitetssjukhuset, Göteborg, Sweden; g Brighton and Sussex Medical School, Brighton, UK

**Keywords:** Breast neoplasms, Endocrine therapy, Older adult

## Abstract

**Background:**

This study reports the detailed management and outcomes of women treated with Primary Endocrine Therapy (PET) in a large prospective UK cohort of older women (≥70) with breast cancer.

**Methods:**

This was an unplanned secondary analysis of a prospective, multicentre, observational study (The Age Gap study). Data were collected at baseline and regular intervals on patient, tumour and treatment characteristics with tumour RECIST response category recorded. Direct study follow-up was 24 months with longer-term survival data obtained from the UK cancer registry.

**Results:**

The Age Gap study recruited 3316 women across 56 breast units. Primary endocrine therapy (PET) was initiated for 505/3316 (15 %) women; median age was 84 (IQR 79–88) with median follow-up 41.9 months (IQR 27–60). Death occurred in 205/505(40.6 %) patients, 160/205; 78 % non- Breast Cancer related, 45/205; 21.9 % Breast Cancer related. Multivariate analysis identified older age (HR-1.055(95 % Confidence Interval: 1.029–1.084); P < 0.001) and higher Charlson Index (HR-1.166 (1.086–1.252); P < 0.001) as risk factors for all-cause mortality, but conversion to surgery (HR-0.372(0.152–0.914); P = 0.031) was protective. Grade 3 cancer (G1 vs G3 HR-0.28 (0.094–0.829); P = 0.022 & G2 vs G3 HR-0.469 (0.226–0.973); P = 0.042), axillary positivity (axilla positivity HR-2.548 (1.321–4.816); P = 0.005) and change of endocrine therapy (HR-3.010 (1.532–5.913); P = 0.001) were associated with worse breast cancer specific survival (BCSS). RECIST category was not significantly associated with either overall survival or BCSS (P > 0.05).

**Conclusion:**

Early disease response and change of endocrine therapy are not significantly associated with overall survival, conversion to surgery is linked to improved outcome. Prognosis is largely determined by age and comorbidity in older women treated with PET.

## Highlights

• For PET patients, prognosis is largely determined by age and comorbidity, as most deaths are not breast cancer related.
• Change of endocrine therapy and early disease response are not significantly associated with overall survival.
• Patients with change of management to surgery do better; this may be due more to innate fitness for surgery than the operation.

## 1 Introduction

The standard of care for the curative treatment of primary, early-stage, invasive breast cancer remains surgery. Selected patients with oestrogen receptor positive (ER + ve) tumours, may be treated with endocrine therapy only – Primary Endocrine Therapy (PET). In a Cochrane review comparing surgery to PET, patients >70 years old have similar 5 year overall survival, although data from longer term follow-up suggests a benefit to surgery with superior local control rates [1,2]. The Bridging the Age Gap Cohort study of older women with breast cancer [3] demonstrated that patients treated with PET had similar breast cancer specific survival outcomes compared to surgery patients at median 52 months follow up [4]. Longer term follow-up of this cohort is due to take place shortly. A recent meta-analysis found that overall survival may be improved with primary surgery compared to PET, although patient selection impacted on outcomes [5].

Current UK and European guidelines recommend PET only in those with significant co-morbidity that precludes surgery [6] or with a short estimated life expectancy (<5 years) [7]. Despite these limited indications, data from the US [8] and the Netherlands [9] suggest PET use in the elderly population may be increasing, and there is wide variation in the use of PET in the UK [10,11] with an increase in use with age, with over 30 % of ER + breast cancers treated without surgery in those aged >80 [12]. Data from women with ER negative cancers suggests that 90 % of older women are able to be treated surgically [12].

Once patients are initiated on PET, monitoring protocols vary substantially. The mean duration of response to PET is 18–24 months [1]. Active patient follow-up allows disease monitoring and, if the disease escapes local control, switching endocrine therapy to a drug with a different mechanism of action or surgery if the patient is able to tolerate this. A survey of UK breast surgeons in 2013 [11] found significant variations in follow up of patients on PET, and deviation from the original studies of PET [[13], [14], [15], [16], [17], [18], [19], [20]]. Qualitative semi-structured interviews performed as part of the Bridging the Age Gap study revealed there were variations in tumour assessment method, assessment interval and surgeon reaction to loss of disease control [21].

A current unknown is whether early response predicts patient outcomes, with respect to overall survival, or whether disease progression (and any subsequent change of management) affects outcomes in this group of patients, who generally have a limited life expectancy.

This study represents an unplanned secondary analysis of a pre-existing large, prospective observational multicentre UK study of the management of breast cancer in older women (>70). The aim of this study is to document patient selection criteria for PET and the impact of local disease response and change of management has on survival outcomes.

## 2 Methods

### 2.1 Data collection

#### 2.1.1 Study design

This is an unplanned sub-study of the Bridging the Age Gap study, a prospective multicentre observational study [22,23]. The study was a pragmatic, observational, non-interventional study, requiring no change to usual clinical practice and patient management.

#### 2.1.2 Research funding, ethics and governance

The study had ethics approval (Integrated Research Application System number: 115550) and each participating site was granted local research governance approval. The study was sponsored by Doncaster and Bassetlaw Teaching Hospitals NHS foundation trust and funded by the UK National Institute for Health Research (NIHR) (Programme Grants for Applied Research number 2012-18RP-PG-1209-10071).

#### 2.1.3 Eligibility criteria

Older women aged ≥70 years with operable breast cancer (TNM stages: T1-3 and operable T4b, N0-1, M0) were eligible for the wider Bridging the Age Gap study, those documented as having Endocrine Therapy as the primary treatment were included in this sub-study.

#### 2.1.4 Recruitment

Prospective recruitment took place at the time of diagnosis from 56 UK units between July 2012–June 2018. Written informed consent was obtained directly from each patient or from a proxy in the case of women with cognitive impairment.

#### 2.1.5 Data collection

Data were collected on baseline health status (age, Charlson Comorbidity Index (CCI) [24], Mini Mental State Examination (MMSE) [25], Activities of Daily Living (ADL) [26], Instrumental Activities of Daily Living (IADL) [27], Eastern Cooperative Oncology Group Performance Status (ECOG-PS), derived frailty score (Rockwood's accumulation of deficits model) and Quality of Life (QoL) scores (using the previously validated EQ-5D [28] questionnaire). Tumour characteristics at baseline were recorded by treating clinician using the local unit's usual diagnostic pathways for primary tumour size (and classified by TNM stage criteria measured clinically or radiologically) and axillary nodal status (positive, negative, unknown; determined by clinical assessment ± ultrasound). Tumour receptor profile was determined according to local laboratory protocols and recorded as oEstrogen Receptor (ER) status (ER strongly positive = Allred score 7–8/8 or H score >200, ER weakly positive = Allred score 3 to 6 or H score 50–200, ER negative = Allred score 0–2 or H score <50 [29]), Progesterone Receptor (PgR) status (positive, negative, unknown) and HER2 status (positive, negative, unknown).

Data were collected at 5 follow-up time points, with some flexibility to assessor and patient (6 weeks, 6 months, 12 months, 18 months and 24 months from baseline assessment).

At each follow up visit patients were assessed by the treating clinician for local recurrence, QoL, adverse events, treatment received and PET adherence (classed as ‘taken all’, ‘taken most’ or ‘rarely/never taken’). Tumour status was assessed with tumour maximum recorded diameter compared to the previous recorded measurement and categorised according to the Response Evaluation Criteria in Solid Tumours (RECIST) criteria (Complete Response- Disappearance of all target lesions; Partial Response - ≥30 % decrease in diameter of target lesion; Progressive Disease - ≥20 % increase in diameter of target lesion; Stable Disease – neither sufficient shrinkage to qualify for Progressive Disease, nor increase to qualify for Partial response) [30]. Analysis of RECIST categories was performed by the ‘worst’ (i.e. closest to (or actual) progression) RECIST category by that timepoint. Missing follow-up data were logged as such in analysis but if a subsequent visit was attended suggesting disease response was maintained it was inferred that the preceding visit was also a response. If progression was noted it was inferred to be first noted at the time of the current visit. Patients who had missing follow up RECIST data at a particular timepoint were excluded from analysis at that time point.

Complications were recorded and categorised using the Common Terminology Criteria for Adverse Events system (CTCAE v4.0).

Longer term survival outcomes were obtained from the UK Cancer Registry (with specific patient consent). Deaths were reviewed by the Chief Investigator blind to treatment decisions and categorised as disease-related if the death was related to the initial breast cancer, or other causes. Patients for whom the cause could not be established were excluded from cause specific analyses.

### 2.2 Outcomes

The primary outcome was all cause mortality. Secondary outcome was breast cancer specific mortality. Stated reason for PET allocation, method of follow-up, rates of RECIST response, change of management and time to treatment failure were recorded.

The reporting of this study conforms to the STROBE guidelines [31] (Supplementary Fig. 1).

### 2.3 Statistical analysis

Database information was managed in Excel (Microsoft) and statistical analysis performed with SPSS v.28 software (IBM), with Kaplan Meier and risk tables completed in Stata SE 18.0 (StatCorp, Texas, USA). Continuous symmetrically-distributed data are summarised with means (standard deviation), skewed data are summarised with medians (IQR) and categorical data as percentages.

Associations between the four RECIST criteria groups (unrecorded, complete/partial response, stable disease, progressive disease) at 6, 12 and 24 months and baseline risk factors were investigated with the chi-squared test (for categorical risk factors) and one-way ANOVA for continuous risk factors.

Survival time analysis was performed according to the worst recorded RECIST criteria at 6, 12 and 24 months. Complete response and partial response categories were combined due to low numbers (<10 patients) with a complete response. The survival endpoint was death or end of study period (March 1, 2020) where data were censored; median survival time and 95 % upper and lower Confidence Intervals are reported for the estimates. Survival was estimated by Kaplan-Meier survival curves and compared between the four RECIST criteria groups (unrecorded, complete/partial response, stable disease, progressive disease) with the generalised log-rank test.

Univariate survival analysis of tumour and patient characteristics was performed with Cox regression analysis. Statistically significant factors, with a *P*-value <0.05 from the univariate analysis, were entered into a multivariate Cox regression model. Hazard ratio and 95 % upper and lower Confidence intervals (CI) are described. Statistical significance was defined as P < 0.05.

## 3 Results

### 3.1 Patient characteristics

**Fig. 1:**
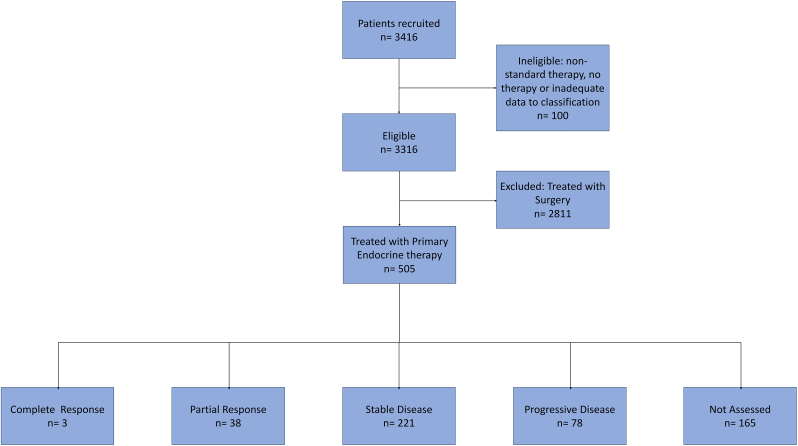
Study flow diagram. RECIST criteria breakdown are patients ‘worst response’ recorded by 12 months.

The parent Age Gap study recruited a total of 3416 patients, of whom 3316 were eligible. Primary endocrine therapy was used for 505 patients (505/3316, 15.2 %)(Fig. 1). Median follow up was 41.9 months (IQR 27–60). Baseline demographics, tumour characteristics and reason for initiation of PET are displayed in Table 1. This demonstrates that 218/505 (43 %) of patients were offered a choice of PET or surgery and chose PET. Endocrine therapy was with an aromatase inhibitor in 89 % (447/505), usually letrozole (414/505, 82 %). Adherence to PET (Compliance) was high, with 445/505 (88.1 %) recording taking the medication ‘all the time,’ although there was no mechanism to confirm adherence. Adverse events were relatively common with 201/505 (39.8 %) of patients reporting side effects at some time during the follow-up period. Attendance at follow-up appointments was variable (between 0 and 5 follow up appointments), as was follow-up imaging (210/505; 41.5 % had subsequent ultrasound, 104/505; 20.6 % subsequent mammogram) (Supplementary Table 1). Overall, 205/505 (40.6 %) patients died (all cause mortality), with breast cancer noted as cause of death for 21.9 % (45/205) of deaths.

**Table 1:** Baseline patient and tumour characteristics, rationale of PET initiation and mortality.

Characteristic		Number with available data	Summary statistic	Number of patients N = 505	%
Age (years)	*Mean(SD)*	505	83.43 (6.49)		
	*Median (IQR)*		84 (79–88)		
CCI	*Median (IQR)*	464	5.5 (4–7)		
EQ-5D	*Mean (SD)*	370	0.76 (0.22)		
ADL	*Median (IQR)*	387	19 (17–20)		
iADL	*Median (IQR)*	404	7 (5–8)		
MMSE	*Median (IQR)*	276	28 (26–29)		
MMSE Categories	*Normal function*			183	36
	*Mild impairment*			71	14
	*Moderate impairment*			21	4
	*Severe impairment*			1	0.2
	*Unknown*			229	45
ECOG	*Fully active*			140	28
	*Ambulatory*			67	13
	*Restricted in physically strenuous activity*			168	33
	*Limited selfcare*			76	15
	*Completely disabled*			7	1.4
	*Unknown*			47	9
Tumour size TNM stage	*1*			195	39
	*2*			271	54
	*3*			27	5
	*unknown*			12	2
Grade	*1*			102	20
	*2*			330	65
	*3*			56	11
	*Unknown*			17	3
ER status	*Strongly Positive*			480	95
	*Weakly Positive*			21	4.1
	*Negative*			2	0.4
	*unknown*			2	0.4
PR status	*Positive*			227	45
	*Negative*			41	8
	*Unknown*			237	47
HER 2 status	*Positive*			32	6
	*Negative*			318	63
	*Unknown*			155	31
Axillary status	*Positive*			89	18
	*Negative unknown*			411	81
	*unknown*			5	1
Reason for initiating PET	*Participant preference after offer of a choice*			218	43
	*Clinician preference at request of participant*			61	12
	*Participant likely to be at increased risk so no surgery offered*			80	16
	*Proxy decision by carers*			11	2
	*Other*			23	5
	*Unknown*			112	22
PET initiated on	*Letrozole*			414	82
	*Anastrozole*			31	6
	*Exemestane*			7	1.4
	*Tamoxifen*			22	4
	*Unknown*			31	6
Breast Cancer specific mortality		205		45	8.9
All cause mortality		505		205	40.6

N – denotes number of patients with the demographic information available for analysis. Continuous data presented as mean (standard Deviation), or median (IQR), discrete data as percentage of total number.CCI – Charlson Comorbidity Index; EQ-5D – EuroQol-5D; ADL – Activities of Daily Living; iADL – Instrumental Activities of Daily Living; MMSE – Mini Mental State Examination; ECOG – Easter Cooperative Oncology Group Performance Status; TNM – Tumour, Node, Metastasis; ER – oEstrogen Receptor; PR – Progesterone Receptor; HER2 – Human Epidermal Growth Factor 2; PET – Primary Endocrine Therapy.

### 3.2 Association of RECIST category with outcomes

The RECIST response category at 12 months did not correlate with the majority of baseline characteristics with the exception of tumour size. Those with stable disease had a significantly higher proportion of T2 tumours, and lower proportion of T1 tumours than those with progressive disease (Stable disease T1 = 31 %; T2 = 64 % vs progressive disease T1 51 %; T2 = 39 %; P < 0.001) (Supplementary Table 2). There was no correlation with degree of ER positivity. Most patients had strongly ER positive tumours (Table 1).

**Fig. 2:**
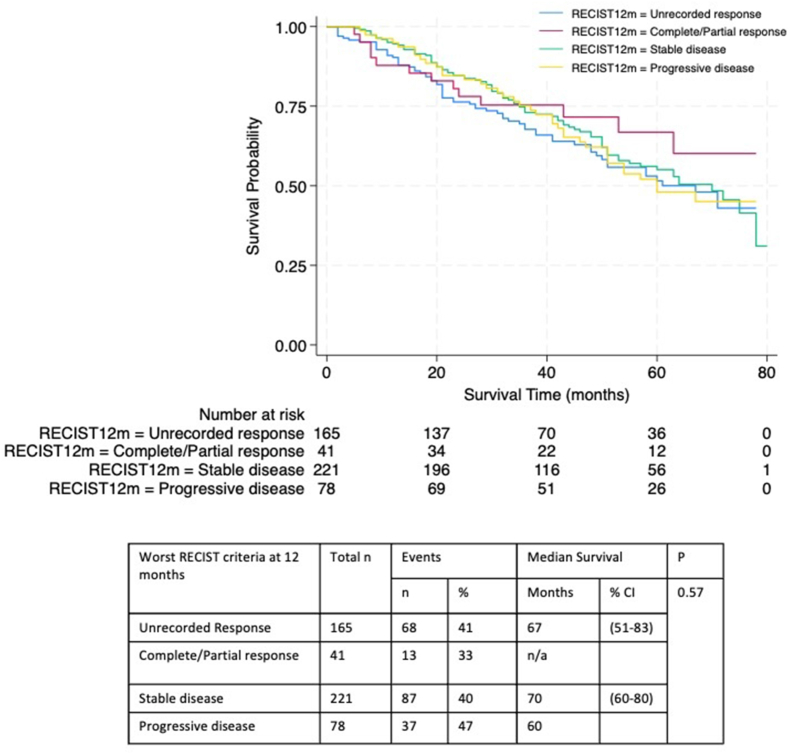
(Colour figure)- Survival Probability of patients according to worst RECIST category recorded at 12 months. Displayed as Kaplan Meier survival curve with number of patients at risk at time points, with number of deaths in parenthesis. Statistical significance P < 0.05; log rank test.

Overall survival analysis was performed according to the RECIST response category at 12 months (Fig. 2) and at 6 and 24 months (Supplementary Fig. 2) to investigate if responses by different timepoints to PET predicted overall survival.

In no timepoint comparison was 50 % mortality reached in any group, limiting calculation of median survival times for comparison. Comparison of survival distribution demonstrated no statistically significant difference between groups according to worst RECIST category at 6, 12 or 24 months (P > 0.05; log rank test).

### 3.3 Patients undergoing a change of management (COM)

Analysis was performed for those who underwent change of endocrine therapy (n = 61) or converted to surgery (n = 35) versus no treatment change (n = 409). Baseline demographics are displayed in Supplementary Table 3. Those who converted to surgery were significantly younger (conversion to surgery median age: 80 years (75–83) versus change of endocrine therapy: 84 years (81–90) versus no treatment change: 84 (79–88), P = 0.003), fitter (ECOG status ‘fully active’ - conversion to surgery 10/35, (29 %) versus change in ET 11/61 (18 %) versus no treatment change 107/409 (26 %), (P < 0.001), and more likely have weakly ER + ve tumours (weakly ER + ve - conversion to surgery 4/35, 11.4 % versus no treatment change 12/409 2.9 %; P = 0.02), but not compared to ET change (5/61, 8.2 %; P > 0.05). There was no statistically significant association between RECIST category and change of management groups (P = 0.037; Table 2).

**Table 2:** Table demonstrating the worst RECIST criteria response rate at 12 months and change of management. There was no significant difference between groups, Chi square test P = 0.37.

		RECIST category				
		n	Complete/Partial response	Stable disease	Progressive disease	Unrecorded response
Change of management	No treatment change	409	34 (8.3 %)	180 (44.0 %)	58 (14.2 %)	137 (33.5 %)
	ET change	61	6 (9.8 %)	24 (39.3 %)	15 (24.6 %)	16 (26.2 %)
	Conversion to Surgery	35	1 (2.9 %)	17 (48.6 %)	5 (14.3 %)	12 (34.3 %)

Time to change of management was less than 6 months in 48/61 (79 %) of ET change and 25/35 (71 %) of conversion to surgery, between 6 and 12 months in 17/61 (27 %) of ET change and 8/35 (23 %) of conversion to surgery, and >12 months in 21/61 (34 %) of ET change and 2/35 (5 %) of conversion to surgery.

Survival analysis and outcomes according to change of management groups (no treatment change, change of PET medication and conversion to surgery) are displayed in Supplementary Fig. 3. Median survival was not significantly different between no treatment change (median survival 64 months (95%CI 55–73)) versus change of PET (median survival 51 months (95%CI 30–72)), but these groups were significantly different to conversion to surgery (50 % mortality not reached; P = 0.006).

### 3.4 Survival analysis

#### 3.4.1 All cause mortality

Cox regression analysis of tumour, patient and treatment related factors was performed (Table 3).

**Table 3:** Table displaying results of cox regression overall survival analysis of patients and relevant baseline characteristics, treatment change and follow up regime. Analysis performed with cox regression analysis, multivariate analysis n = 464.

Characteristic	N	Univariate			Multivariate		
		HR	CI	P	HR	95 % CI	P
Age	505	1.08	1.06–1.11	<0.001^a^	1.06	1.03–1.08	<0.001^a^
Charlson Index	464	1.23	1.16–1.31	<0.001^a^	1.17	1.08–1.25	<0.001^a^
Grade (vs G3)	488			0.36			
1		0.81	0.51–1.31	0.40			
2		0.74	0.49–1.11	0.15			
3		–		–			
Tumour size	493	1.0	0.99–1.01	0.76			
ER status	503	0.81	0.47–1.41	0.46			
PGR status	268	0.73	0.42–1.25	0.25			
							
HER2 status	350	0.97	0.52–1.79	0.97			
Axilla + ve	505	0.97	0.66–1.44	0.89			
Treatment change (vs no change)				0.011^a^			0.092
- PET change		1.22	0.82–1.81	0.33	1.02	0.65–1.57	0.95
- Surgery		0.32	0.14–0.71	0.005	0.37	0.15–0.91	0.029^a^
Worst RECIST at 12 months (vs unrecorded response)	505			0.56			
- Complete/Partial response		0.69	0.38–1.24	0.22			
- Stable disease		0.86	0.62–1.17	0.32			
- Progressive disease		0.91	0.61–1.36	0.65			

CCI = Charlson Comorbidity Index, HR = Hazard Ratio, CI = 95 % Confidence Interval, Wald statistic and P value.

a significantly different: P < 0.05.

On univariate analysis significant risk factors associated with all cause mortality were older age (HR 1.08 (1.06–1.11); P < 0.001), and higher Charlson Co-morbidity Index (HR 1.23 (1.16–1.31); P < 0.001), whereas conversion to surgery was associated with a reduction in mortality (HR 0.32 (0.14–0.71); P = 0.005).

On multivariate analysis of all cause mortality older age (HR 1.06 (1.03–1.08); P < 0.001), higher Charlson Co-morbidity Index (HR 1.17 (1.09–1.25); P < 0.001) and conversion to surgery (HR 0.37 (0.15–0.91); P = 0.029) all remained statistically significant.

##### 3.4.1.1 Breast cancer specific mortality

Similar analysis was performed for breast cancer specific survival (Table 4). On univariate cox regression the baseline tumour demographics of higher grade, positive axillary nodal status, and a change of endocrine therapy were associated with a significant impact on breast cancer specific mortality. These differences remained on multivariate analysis: Grade 3 cancer (G1 versus G3 HR 0.28 (0.094–0.83); P = 0.022 & G2 vs G3 HR 0.47 (0.23–0.97); P = 0.042) and axillary positivity (axilla positivity HR 2.55 (1.32–4.82) P = 0.005) were associated with a significant increase in breast cancer specific survival. Change of endocrine therapy was associated with a signficantly increased breast cancer specific mortality (HR 3.01 (1.53–5.91) P = 0.001), with conversion to surgery demonstrating a non significant trend towards reduced breast cancer specific mortality, (HR 0.66(0.19–2.26) P = 0.51).

**Table 4:** Table displaying results of cox regression breast cancer specific survival analysis of patients and relevant baseline characteristics, treatment change and follow up regime. Analysis performed with cox regression analysis. Multivariate analysis n = 488.

Characteristic	N	Univariate			Multivariate		
		HR	CI	P	HR	95 % CI	P
Age	505	1.04	0.99–1.10	0.076			
Charlson Index	464	1.14	0.98–1.33	0.087			
Grade (vs G3)	488			0.007^a^			0.043^a^
1		0.22	0.076–0.61	0.004^a^	0.22	0.094–0.83	0.022^a^
2		0.38	0.19–0.74	0.005^a^	0.47	0.23–0.97	0.042^a^
3		–					
Tumour size	493	1.00	0.99–1.01	0.72			
ER status	503	0.52	0.21–1.26	0.20			
PGR status	268	1.42	0.52–3.89	0.50			
HER2 status	350	0.67	0.20–2.25	0.52			
Axilla + ve	505	2.60	1.38–4.89	0.003^a^	2.55	1.32–4.82	0.005^a^
Treatment change (vs no change)	505			0.004^a^			0.002
- ET change		3.31	1.72–6.38	<0.001^a^	3.010	1.53–5.91	0.001^a^
- Surgery		0.83	0.27–2.90	0.83	0.660	0.19–2.26	0.51
Worst RECIST at 12 months (vs Unrecorded response)				0.14			
Complete/Partial response		0.00	0.00->10	0.97			
- Stable disease		0.70	0.36–1.34	0.29			
- Progressive disease		0.95	0.43–2.08	0.90			

Non-breast cancer deaths were censored at the date of death. CCI = Charlson Comorbidity Index, HR = Hazard Ratio, CI = 95 % Confidence Interval, Wald statistic and P value

a significantly different: P < 0.05.

## 4 Discussion

This study presents data from a large contemporary UK cohort of patients treated with PET. In the small number who had conversion to surgery there was significant association with improved overall survival. Early tumour response (either response or progression) and a change of endocrine therapy were not associated with overall survival outcomes. These findings suggest that in patients who will not have surgery whilst on PET, change of endocrine therapy in response to disease progression may not have any clear survival benefit. For the majority of these women, death due to non-breast cancer causes is the greater risk and local control may be achieved by change to alternate endocrine therapy. Likewise, it suggests that for women who are fit for surgery, surgery may be beneficial.

This study reports the reason for initiating PET. The most common reason given was patient preference after being offered a choice by their clinician (47 %). This reflects our previous findings that when a comparable population of patients are given more information about PET in the form of a decision support tool, they are more likely to choose PET over surgery [32], which could be due to prioritising quality of life [33,34]. Adherence to prescribed PET was higher than in the adjuvant setting, with 88.1 % reporting adherence to their medication [35] with a relatively low burden of side effects (39.8 % experienced any AEs) [36,37]. This could be due to the perceived importance of PET as the primary cancer treatment, or increased pre-existing medication increasing adherence [35].

An interesting finding is that over 50 % of this population was still alive after a median 42 months of follow-up suggesting that breast surgeons frequently underestimate life expectancy in this patient group [11].

Follow-up methodology (clinical, US or mammographic) for patients on PET is varied -almost half (46 %) of patients had no imaging during follow-up, in keeping with a recent survey [11], but very different to the early PET trials [17].

Our results have confirmed previous findings that higher tumour grade and axillary involvement are predictors of response to PET and are associated with worse breast cancer specific survival [38,39]. The significance of tumour size in predicting tumour response is conflicting in the literature [38,39], and in our series was not a predictor of worse breast cancer specific survival.

The impact of different RECIST response outcomes was not clear, which may reflect smaller numbers in certain groups (complete response and progressive disease) compounded by high levels of missing data. There was no statistically significant association between RECIST category at any timepoint and OS. This may be because, in this more elderly and comorbid population, early disease response predicts the expected trajectory of breast cancer, but death from other causes intervenes and so the variation in breast cancer prognosis by early disease response is not observed. This is exemplified by the finding that less than 25 % of deaths were breast cancer related.

Previous literature investigating endocrine therapy for advanced primary breast cancer found that 5 year overall survival was significantly poorer in those with progressive disease at 6 months [[40], [41], [42]]. The prognostic significance of early response in our study population which is older and with smaller baseline tumour size (i.e. not advanced) is much more limited.

Another motivation for monitoring tumour size is to identify disease progression and change management.

In this study, the 35 patients with a change of management to surgery had a better survival than other treatment groups, in keeping with previous literature [1]. These patients were younger, and with better functional status than other treatment groups suggesting that selection bias may account for the apparent survival benefit. Previous propensity matching of PET and surgery groups suggests no survival difference in the least fit group in whom PET is indicated [43]. The majority (71 %) underwent surgery within 6 months of commencing endocrine therapy (ET). It is possible that some of these patients were having planned neoadjuvant endocrine therapy.

The subgroup of patients who had ET therapy changed from one endocrine agent to another had a greater proportion of progressive disease – a trend that has been previously reported [44]. It could be that patients progressed and without this change of management their survival would have been worse. Importantly, ET change within the first 2 years was not associated with worse OS.

It is therefore not clear that early disease response is associated with OS in this cohort, but progression after 24 months may be. In light of the known slow trajectory of ER positive breast cancer longer follow-up will be required.

## 5 Limitations

One third of patients (165/505) had either one or no tumour measurement recorded and thus could not be given a RECIST category. In addition, infrequency of tumour measurements precluded any observations regarding COM as a response to disease progression which was why the worst RECIST category achieved by a given timepoint was used. Follow-up appointments and tumour measurement assessment method was variable which meant those more intensely followed up may be overrepresented. Patients with fewer than two tumour measurements were included in analysis as ‘unrecorded’. These patients overall survival outcomes were similar to the RECIST subgroups. This ‘limitation’ therefore gives valuable insight into the outcomes of patients with minimal follow up (a group underreported in previous studies), and that outcomes may not be affected by observation.

This study only provides insight on the impact of early clinical activity within the first 24 months after commencing PET on survival and the impact of change of management and locaregional progression after 2 years cannot be analysed in this dataset.

## 6 Conclusion

The main findings from this current analysis of a previously described cohort is that for patients with early invasive breast cancer treated with primary endocrine therapy alone (93 % of this study population), change of endocrine therapy and early disease response are not significantly associated with overall survival. Prognosis is largely determined by age and comorbidity for this group of older, less fit patients where the majority of deaths are not related to the breast cancer. Tumour stage, including nodal status and grade, are key prognostic factors for BCSS. The role of monitoring type (clinical, ultrasound) for early disease progression is unclear. Patients with an early change of management to surgery do better but this may relate more to their innate fitness for subsequent surgery rather than to the protective effect of the surgery itself.

## Conflict of interest

The authors declare no conflict of interest.

## Funding source

This paper presents independent research funded by the 10.13039/501100000272National Institute for Health Research (NIHR) under its Programme Grants for Applied Research Programme (Grant Reference Number RP-PG-1209-10071). The views expressed are those of the authors and not necessarily those of the NIHR or the Department of Health and Social Care.

TH is funded by an National Institute of Health research Academic Clinical Lectureship

## CRediT authorship contribution statement

**Thomas Hubbard:** Writing – review & editing, Writing – original draft, Methodology, Formal analysis, Conceptualization. **Georgia Wright:** Writing – review & editing, Writing – original draft, Methodology, Formal analysis, Conceptualization. **Jenna Morgan:** Writing – review & editing, Supervision, Investigation, Conceptualization. **Charlene Martin:** Writing – review & editing, Investigation. **Stephen Walters:** Writing – review & editing, Investigation. **Kwok-Leung Cheung:** Writing – review & editing, Investigation. **Riccardo Audisio:** Writing – review & editing, Investigation. **Malcolm Reed:** Writing – review & editing, Investigation. **Lynda Wyld:** Writing – review & editing, Writing – original draft, Visualization, Validation, Supervision, Resources, Project administration, Methodology, Investigation, Funding acquisition, Formal analysis, Data curation, Conceptualization.
