# Supplementary material for: Management and outcomes for older women with early breast cancer treated with primary endocrine therapy (PET)
Source: Breast. 2024 Jul 8;77:103768. doi: 10.1016/j.breast.2024.103768 (PMC11301393; doi:10.1016/j.breast.2024.103768)
Supplement: Multimedia component 1 [file mmc1.docx]

Supplementary Files

**Supplementary tables 1** *– Follow up schedules and adverse events*

|  | | **Not Intensive Follow up**  **N= 262** | **Intensive Follow up**  N=243 |
| --- | --- | --- | --- |
| *Any Follow up Ultrasound* | % | 43.1% (113/262) | 39.9% (97/243) |
| *Any Follow up mammogram* | % | 24.4% (64/262) | 16.5% (40/243) |

Supplementary table 1.1. Table displaying variation in follow up attended and imaging undertaken. Intensive follow up = recorded follow up appointment at all possible time points while alive; not intensive follow up = missing at least one potential follow up appointment at a possible time point while alive, Of these 86/262 (17%) missed one potential follow up appointment, 72 (14%) missed two, 56 (11%) missed three, and 48 (9.5%) missed four.

|  | % with Aes |
| --- | --- |
| Week 6 | 27.54% |
| Month 6 | 28.22% |
| Month 12 | 20.62% |
| Month 18 | 18.97% |
| Month 24 | 17.02% |

*Supplementary table 1.2, Percentage of patients who attended each appt that reported any adverse effect of endocrine therapy*

|  | Week 6 | Month 6 | Month 12 | Month 18 | Month 24 |
| --- | --- | --- | --- | --- | --- |
| Hot flushes | 25.00% | 32.14% | 37.50% | 28.21% | 25.00% |
| Asthenia | 16.67% | 27.78% | 33.33% | 30.56% | 31.82% |
| Joint pain | 37.50% | 36.96% | 42.86% | 23.68% | 25.00% |
| Vaginal dryness | 32.43% | 34.72% | 44.44% | 23.08% | 42.86% |
| Hair thinning | 33.85% | 29.41% | 22.22% | 40.91% | 40.85% |
| Rash | 30.56% | 34.69% | 33.33% | 28.57% | 46.15% |
| Nausea | 25.00% | 38.10% | 21.74% | 32.69% | 25.00% |
| Diarrhoea | 38.89% | 40.00% | 18.18% | 46.15% | 37.50% |
| Headache | 36.00% | 30.23% | 40.00% | 50.00% | 34.21% |
| Vaginal bleeding | 34.00% | 32.79% | 24.00% | 40.00% | 26.67% |
| Vomiting | 28.13% | 32.14% | 47.83% | 36.36% | 31.25% |
| Somnolence | 30.77% | 28.00% | 32.35% | 30.77% | 36.11% |
|  |  |  |  |  |  |

*Supplementary table 1.3, Percentage of patients who attended each appt that reported each specific adverse effect.*

|  | Week 6 | Month 6 | Month 12 | Month 18 | Month 24 |
| --- | --- | --- | --- | --- | --- |
| % 'Taken all' | 96.19% | 94.43% | 93.33% | 93.94% | 95.38% |
| % 'Taken most' | 3.33% | 3.98% | 5.33% | 4.76% | 4.62% |
| % 'Rarely / never taken' | 0.40% | 0.20% | 0.40% | 0.20% | 0.00% |

*Supplementary table 1.4, Percentage of patients who attended each appt that reported each compliance category.*

**Supplementary Table 2 -** *Baseline characteristics by RECIST category at 12 months*

|  | | **Worst RECIST category at 12 months** | | | | |
| --- | --- | --- | --- | --- | --- | --- |
|  |  | **Complete response** | **Partial Response** | **Stable disease** | **Progression** | **Not recorded** |
|  |  | N =3 | N= 38 | N =221 | N= 78 | N=165 |
| **Age (years)** | Median | 80 (72-80) | 84 (78-90) | 85 (81-89) | 83 (79-87) | 83 (77-87) |
| **Modified CCI score** | Median | 6 (5-7) | 6 (3-17)) | 5 (3-13) | 5 (3-10) | 6 (3-15) |
| **Tumour TNM T stage** | 1 | 0 | 10 (26%) | 68 (31%)* | 40 (51%)* | 77 (47%) |
|  | 2 | 3 (100%) | 26 (68%) | 142 (64%)* | 30 (39%)* | 7 (42) |
|  | 3 | 0 | 2 (5%) | 10 (5%) | 7 (9%) | 8 (5%) |
|  | Unknown | 0 | 0 | 1 (0.5%) | 1 (1%) | 10 (6%) |
| **Grade** | 1 | 1 (33%) | 5 (13%) | 39 (17%) | 14 (18%) | 43 (26%) |
|  | 2 | 2 (67%) | 30 (79%) | 148 (67%) | 49 (63%) | 101 (61%) |
|  | 3 | 0 | 2 (5.3%) | 25 (11%) | 12 (16%) | 17 (10%) |
|  | Unknown | 0 | 1 (2.6%) | 9 (4%) | 3 (4%) | 4 (2.4%) |
| **ER status** | Strongly Positive | 3 (100%) | 36 (94.7%) | 216 (97.7%) | 71 (91%) | 154 (93.3%) |
|  | Weakly Positive | 0 | 2 (5.3%) | 4 (0.8%) | 7 (9%) | 8 (4.8%) |
|  | Negative | 0 | 0 | 0 | 0 | 2 (1.2%) |
|  | Unknown | 0 | 0 | 1 (0.5%) | 0 | 1 (0.6%) |
| **PR status** | Positive | 2 (66%) | 18 (47%) | 38 (49%) | 38 (48%) | 66 (40%) |
|  | Negative | 0 | 5 (13%) | 21 (10%) | 4 (5%) | 11 (7%) |
|  | Unknown | 1 (33%) | 15 (40%) | 97 (44%) | 36 (46%) | 88 (53%) |
| **HER2** | Positive | 0 | 0 | 14 (6%) | 7 (9%) | 11 (7%) |
|  | Negative | 2 (67%) | 25 (66%) | 138 (43%) | 47 (60% | 106 (64%) |
|  | Unknown | 1 (33%) | 13 (34%) | 69 (31%) | 24 (31%) | 48 (29%) |
| **Axilla** | Positive | 1 (33%) | 4 (11%) | 33 (15%) | 12 (15%) | 23 (14%) |
|  | Negative/unknown | 2 (66%) | 34 (90%) | 188 (85%) | 66 (85%) | 142 (86%) |

Supplementary Table 2- Baseline characteristics of patients grouped according to the RECIST category recorded at 12 months. There was no significant difference between groups , Chi square test for categorical categories, median test (k samples) for non-parametric data; *=statistically significant difference between groups P=0.05

**Supplementary Table 3** – *Baseline characteristics by Change of Management group*

|  | | **Changed Either Drug or Conversion to Surgery** | | | | **No Treatment change** |  | P value |
| --- | --- | --- | --- | --- | --- | --- | --- | --- |
|  |  |  |  |  |  |  | n |  |
|  |  | **ET change** | n | **Surgery** | n | N=409 |  |  |
|  |  | N=61 |  | N=35 |  |  |  |  |
| **Age (years)** | Median | 84 (81-90)* | 61 | 80 (75-83)* | 35 | 84 (79-88)* | 409 | *P=0.003 |
| **Modified CCI score** | Median | 6 (4-7, 3-15) | 51 | 5 (4-6, 3-11) | 34 | 6 (4-7, 3-17) | 379 | Ns |
| **ECOG performance status** | Fully Active | 11 (18%)*+ |  | 10 (29%)*+ |  | 107 (26%)*+ |  | *+<0.001 |
|  | Ambulatory | 4 (7%) |  | 1 (3%) |  | 62 (15%) |  | Ns |
|  | Restricted in physically strenuous activity | 23 (38%) |  | 10 (29%) |  | 135 (33% |  | Ns |
|  | Capable of limited self care only | 12 (20%) |  | 1 (3%) |  | 63 (15%) |  | Ns |
|  | Severely disabled | 0 |  | 0 |  | 7 (2%) |  | Ns |
|  | Unknown | 11 (2%) |  | 1 (0.2%) |  | 35 (7%) |  | Ns |
| **TNM tumour size** | 1 | 21 (34%) |  | 16 (8%) |  | 158 (39%) |  | Ns |
|  | 2 | 31 (51%) |  | 18 (51%) |  | 222 (54%) |  |  |
|  | 3 | 6 (10%) |  | 1 (3%) |  | 20 (5%) |  |  |
|  | Unknown | 3 (5%) |  | 0 |  | 9 (2%) |  |  |
| **Grade** | 1 | 14 (23%) |  | 4 (12%) |  | 84 (21%) |  | Ns |
|  | 2 | 38 (62%) |  | 21 (60%) |  | 271 (66%) |  | Ns |
|  | 3 | 9 (15%) |  | 8 (23%) |  | 39 (10%) |  | Ns |
|  | Unknown | 0 |  | 2 (6%) |  | 15 (4%) |  | Ns |
| **ER status (Allred)** | Strongly ER+ve | 56 (91.8%) |  | 30 (85.7%)** |  | 394 (96.3%)** |  | **P=0.02 |
|  | Weaker ER+ve | 5 (8.2%) |  | 4 (11.4%)** |  | 12 (2.9%)** |  |  |
|  | Negative | 0 |  | 0 |  | 2 (0.5%) |  | ns |
|  | Unknown | 0 |  | 1 (2.9%) |  | 1 (0.2%) |  |  |
| **PR status** | Positive | 27 (44%) |  | 17 (49%) |  | 183 (45%) |  | ns |
|  | Negative | 1 (2%) |  | 8 (23%) |  | 32 (8%) |  |  |
|  | Unknown | 33 (54%) |  | 10 (29%) |  | 194 (47%) |  |  |
| **HER2** | Positive | 4 (7%) |  | 4 (11%) |  | 24 (6%) |  | ns |
|  | Negative | 31 (51%) |  | 22 (63%) |  | 265 (65%) |  |  |
|  | Unknown | 26 (43%) |  | 9 (26%) |  | 120 (30%) |  |  |
| **Axilla** | Positive | 6 (10%) |  | 6 (17%) |  | 61 (15%) |  | ns |
|  | Negative/unknown | 55 (90%) |  | 29 (83%) |  | 348 (85%) |  |  |

Supplementary Table 3 – baseline demographics of patients grouped according to change of management groups.

* - significant difference; median test (k samples) with Bonferroni correction; PET vs Surgery (P=0.005); Surgery vs no treatment change (P=0.002); PET vs no treatment change ; ns P>0.05.

*+ - significant difference; Chi square test; Surgery vs PET + Surgery vs no treatment change (P<0.001)

**= Chi Square test Surgery vs no treatment change P=0.02

**Supplementary Figure 1** - *STROBE Statement—checklist of items that should be included in reports of observational studies*

|  | Item No. | Recommendation | Page  No. | Relevant text from manuscript |
| --- | --- | --- | --- | --- |
| **Title and abstract** | 1 | (*a*) Indicate the study’s design with a commonly used term in the title or the abstract | 1 |  |
|  |  | (*b*) Provide in the abstract an informative and balanced summary of what was done and what was found | 3-4 |  |
| Introduction | | | |  |
| Background/rationale | 2 | Explain the scientific background and rationale for the investigation being reported | 5-6 |  |
| Objectives | 3 | State specific objectives, including any prespecified hypotheses | 6 |  |
| Methods | | | |  |
| Study design | 4 | Present key elements of study design early in the paper | 7-8 |  |
| Setting | 5 | Describe the setting, locations, and relevant dates, including periods of recruitment, exposure, follow-up, and data collection | 7-8 |  |
| Participants | 6 | (*a*) *Cohort study*—Give the eligibility criteria, and the sources and methods of selection of participants. Describe methods of follow-up  *Case-control study*—Give the eligibility criteria, and the sources and methods of case ascertainment and control selection. Give the rationale for the choice of cases and controls  *Cross-sectional study*—Give the eligibility criteria, and the sources and methods of selection of participants | 7 |  |
|  |  | (*b*) *Cohort study*—For matched studies, give matching criteria and number of exposed and unexposed  *Case-control study*—For matched studies, give matching criteria and the number of controls per case |  |  |
| Variables | 7 | Clearly define all outcomes, exposures, predictors, potential confounders, and effect modifiers. Give diagnostic criteria, if applicable | 9 |  |
| Data sources/ measurement | 8* | For each variable of interest, give sources of data and details of methods of assessment (measurement). Describe comparability of assessment methods if there is more than one group | 7-9 |  |
| Bias | 9 | Describe any efforts to address potential sources of bias | 7-8 | Prospective, performed by RECIST criteria |
| Study size | 10 | Explain how the study size was arrived at | 7 | Unplanned sub-group analysis. Study size was decided by main study |

Continued on next page

| Quantitative variables | 11 | Explain how quantitative variables were handled in the analyses. If applicable, describe which groupings were chosen and why | 8 | RECIST categories and Change of management explained |
| --- | --- | --- | --- | --- |
| Statistical methods | 12 | (*a*) Describe all statistical methods, including those used to control for confounding | 9-10 |  |
|  |  | (*b*) Describe any methods used to examine subgroups and interactions | 9-10 | Cox regression uni and multi variate analysis |
|  |  | (*c*) Explain how missing data were addressed | 8 | Missing RECIST data was recorded as ‘unrecorded’ and patients included in analysis |
|  |  | (*d*) *Cohort study*—If applicable, explain how loss to follow-up was addressed  *Case-control study*—If applicable, explain how matching of cases and controls was addressed  *Cross-sectional study*—If applicable, describe analytical methods taking account of sampling strategy | 8-9 | Pragmatic study, follow up not dictated, opportunity for data collection at stated timepoints |
|  |  | (*e*) Describe any sensitivity analyses | n/a |  |
| Results | | | | |
| Participants | 13* | (a) Report numbers of individuals at each stage of study—eg numbers potentially eligible, examined for eligibility, confirmed eligible, included in the study, completing follow-up, and analysed | 11-12 | Figure 1 |
|  |  | (b) Give reasons for non-participation at each stage | 12 |  |
|  |  | (c) Consider use of a flow diagram | 12 |  |
| Descriptive data | 14* | (a) Give characteristics of study participants (eg demographic, clinical, social) and information on exposures and potential confounders | 13-15 | Table 1 |
|  |  | (b) Indicate number of participants with missing data for each variable of interest | 13-15 | table 1 |
|  |  | (c) *Cohort study*—Summarise follow-up time (eg, average and total amount) | 11 |  |
| Outcome data | 15* | *Cohort study*—Report numbers of outcome events or summary measures over time | 15-20 |  |
|  |  | *Case-control study—*Report numbers in each exposure category, or summary measures of exposure |  |  |
|  |  | *Cross-sectional study—*Report numbers of outcome events or summary measures |  |  |
| Main results | 16 | (*a*) Give unadjusted estimates and, if applicable, confounder-adjusted estimates and their precision (eg, 95% confidence interval). Make clear which confounders were adjusted for and why they were included | 21-24 | Table 3 +4 |
|  |  | (*b*) Report category boundaries when continuous variables were categorized |  |  |
|  |  | (*c*) If relevant, consider translating estimates of relative risk into absolute risk for a meaningful time period |  |  |

Continued on next page

| Other analyses | 17 | Report other analyses done—eg analyses of subgroups and interactions, and sensitivity analyses |  | All other tables and supplementary data |
| --- | --- | --- | --- | --- |
| Discussion | | | | |
| Key results | 18 | Summarise key results with reference to study objectives | 23 |  |
| Limitations | 19 | Discuss limitations of the study, taking into account sources of potential bias or imprecision. Discuss both direction and magnitude of any potential bias | 23 |  |
| Interpretation | 20 | Give a cautious overall interpretation of results considering objectives, limitations, multiplicity of analyses, results from similar studies, and other relevant evidence | 21-25 |  |
| Generalisability | 21 | Discuss the generalisability (external validity) of the study results | 22-25 |  |
| Other information | |  | | |
| Funding | 22 | Give the source of funding and the role of the funders for the present study and, if applicable, for the original study on which the present article is based | 24 |  |

*Give information separately for cases and controls in case-control studies and, if applicable, for exposed and unexposed groups in cohort and cross-sectional studies.

**Note:** An Explanation and Elaboration article discusses each checklist item and gives methodological background and published examples of transparent reporting. The STROBE checklist is best used in conjunction with this article (freely available on the Web sites of PLoS Medicine at http://www.plosmedicine.org/, Annals of Internal Medicine at http://www.annals.org/, and Epidemiology at http://www.epidem.com/). Information on the STROBE Initiative is available at www.strobe-statement.org.

**Supplementary Figure 2 – Association of RECIST category at 6 and 24 months with outcomes**

**A**

**B**

Supplementary Figure 2 (Colour figure)- Survival Probability of patients according to worst RECIST category recorded at A. 6 months and B. 24 months. Displayed as Kaplan Meier survival curve with number of patients at risk at time points, with number of deaths in parenthesis. Statistical significance P<0.05; log rank test.

**Supplementary figure 3** – *Survival outcomes by Change of Management Group*

|  | No Treatment change  N= 409 | PET change  N= 61 | Surgery change  N= 35 |
| --- | --- | --- | --- |
| Breast cancer mortality | 29 (7.1%) | 13 (21.3%)+ | 3 (8.6%) |
| All cause mortality | 170 (42%) | 29 (47%) | 6 (17%)* |

Supplementary figure 2, Table 1 - showing mortality outcomes according to change of management group. Raw numbers (percentage)

*-significant different comparison of surgery change versus no treatment change and PET change

+ - significant different comparison of PET change vs No treatment change and surgery change; Chi square test P>0.05

Supplementary Figure 2, Figure 1- Cumulative overall survival of patients according to change of management group displayed as Kaplan Meier survival curve.*= significantly different: P<0.05;log rank test,
